# Pregnancy outcomes of a joint obstetric and rheumatology clinic in a tertiary centre: a 2-year retrospective study of 98 pregnancies

**DOI:** 10.1093/rap/rkac026

**Published:** 2022-03-28

**Authors:** Ryan Malcolm Hum, Trixy David, Yen June Lau, Hajira Iftikhar, Sue Thornber, Louise Simcox, Ian Bruce, Clare Tower, Pauline Ho

**Affiliations:** 1 The Kellgren Centre for Rheumatology, Manchester Royal Infirmary, Manchester University NHS Foundation Trust; 2 NIHR Manchester Biomedical Research Centre; 3 Centre for Musculoskeletal Research, The University of Manchester; 4 Saint Mary’s Hospital, Manchester University NHS Foundation Trust; 5 Maternal and Fetal Health Research Centre, The University of Manchester, Manchester, UK

**Keywords:** inflammatory rheumatic diseases, SLE, pregnancy, maternal outcomes, fetal outcomes

## Abstract

**Objectives:**

The purpose of this study was to describe the maternal and fetal outcomes in patients with inflammatory rheumatic diseases attending a joint rheumatology and obstetric clinic in the UK.

**Methods:**

Electronic records of 98 patients attending the joint rheumatology and obstetric clinic between January 2018 and January 2020 were analysed. Data on patient demographics, characteristics (including age, ethnicity, diagnosis, and medications taken during pregnancy), pregnancy outcomes (miscarriage, stillbirth or live birth), maternal complications [infection, post-partum haemorrhage (PPH) or pre-eclampsia] and fetal complications (sepsis, congenital heart block, prematurity and low birth weight) were tabulated. Subgroups of patients based on maternal diagnosis, medications and Ro/La antibody status were described in a similar manner.

**Results:**

The cohort was found to be predominantly Caucasian women >30 years of age, diagnosed with a CTD. Of 98 pregnancies, 97% (*n* = 95) resulted in a live birth, with only 2% resulting in miscarriage (*n* = 2) and 1% in stillbirth (*n* = 1). The median duration of gestation was 38 (interquartile range 37–39) weeks, and the majority of patients had a normal vaginal delivery (35%, *n* = 34), whereas 30% had emergency Caesarean sections (*n* = 29). The median birth weight was 3120 (interquartile range 2690–3410) g. The most common maternal complications were PPH (56%, *n* = 54) and infection (22%, *n* = 21). The most common fetal complications were prematurity (23%, *n* = 22) and low birth weight (17%, *n* = 16).

**Conclusion:**

We report favourable outcomes from this service model, including a high live birth rate, a low miscarriage rate and a high median birth weight. With limited reported data of pregnancy outcomes from joint obstetric/rheumatology clinics, this service model might be beneficial in other centres.

Key messages

- Joint obstetric/rheumatology clinics might be beneficial to improve pregnancy outcomes for patients with inflammatory rheumatic diseases.
- Risk of congenital heart block remains important in the management of Ro/La-positive patients.
- Further data on the safety of newer immunomodulatory drugs in pregnancy are necessary.

## Introduction

Inflammatory rheumatic diseases (IRDs) frequently affect women of childbearing age. Although some studies report improvements in disease activity during pregnancy for certain inflammatory rheumatic diseases (IRDs), active rheumatic disease is frequently associated with adverse pregnancy outcomes (APOs), including maternal complications, such as post-partum haemorrhage (PPH), antenatal infections and pre-eclampsia, and fetal complications, such as miscarriage, stillbirth, prematurity, low birth weight, congenital heart block (CHB) and sepsis [1, 2].

Studies of SLE and inflammatory arthropathies, such as RA, have shown an increased burden of APOs, and therefore management of pregnancy in women with IRDs typically involves optimizing care pre-conception and antenatally to minimize the risk of APOs [3–7].

A key issue is achieving stable disease activity pre-conception and antenatally. Active disease is associated with APOs, and therefore the use of DMARDs to minimize disease activity is essential. However, there exist numerous safety concerns and limited evidence-based recommendations for the prescription of immunomodulatory drugs in pregnancy [8, 9]. To address the need for robust data on pregnancy outcomes and trajectories in women with IRDs, the EULAR Task Force has published recommendations for core data sets for pregnancy registries in rheumatology [10].

From a pragmatic point of view, the clinical care of women with IRDs antenatally requires specialized clinical services that can implement the latest recommendations in order to mitigate the risk of APOs. Antenatal monitoring by a rheumatologist and an obstetrician is recommended, including frequent obstetric and rheumatology reviews, regular blood work and fetal US [3].

The lupus in pregnancy scanning (LIPS) clinic, a weekly joint obstetrics and rheumatology clinic, was established at Saint Mary’s Hospital, Manchester, UK for women with IRDs, especially SLE. The clinic is led by experienced rheumatologists and obstetricians with special interests in IRDs. The clinic accepts referrals from general practitioners (GPs), rheumatologists and other hospital specialists for patients with any IRDs. Accepting referrals for patients with any IRDs as opposed to SLE alone is a unique feature of this service compared with other joint rheumatology and obstetric clinics in other centres. Patients can be reviewed pre-conception and antenatally. Patients with particularly complex conditions and significant risk of APOs are reviewed regularly to term in the LIPS clinic, whereas more straightforward cases are sometimes seen once, with advice given to be followed up by their primary obstetric and rheumatology teams.

The aim of this study was to describe the maternal and fetal outcomes in patients attending a joint obstetric and rheumatology clinic in a tertiary centre in the UK.

## Methods

### The LIPS clinic set-up

The LIPS clinic runs weekly at a tertiary centre in Manchester, UK, and is run by a consultant obstetrician and rheumatologist along with other specialist doctors and midwives. Patients can be seen pre-conception and antenatally and can be referred from throughout the region by GPs, by rheumatologists or by hospital-based physicians in other specialties. Patients can be referred at any stage of pregnancy, but referral pre-conception or as early as possible after pregnancy is confirmed are encouraged in order that patients can be assessed and optimized to improve the outcome.

During appointments, patients undergo obstetric US monitoring, in addition to laboratory investigations and face-to-face consultations. The clinic can see patients for follow-up, typically on a fortnightly or monthly basis, depending on the gestation, the complexity of the case and the availability of appointments in the clinic. When appropriate, patients can also be discharged back to their primary rheumatology and obstetric teams with advice.

### Patients

Electronic records of patients who attended the LIPS clinic between January 2018 and January 2020 were reviewed retrospectively. Patients who had at least one antenatal LIPS clinic visit and who had completed their pregnancy were included for analysis. Patients who were deemed low risk and discharged from the LIPS clinic back to their primary rheumatology and obstetric teams with advice were excluded.

### Patient characteristics

Patient characteristics, including age, diagnosis, ethnicity, disease activity, autoantibody status and medications taken during pregnancy, were tabulated. Clinician diagnosis and assessment of disease activity was used. Disease activity was recorded as being uncontrolled or stable, where uncontrolled disease indicates uncontrolled disease at any point during the pregnancy. Laboratory reference values were used to assess antibody positivity.

### Patient cohorts and sub-cohorts

An LIPS cohort, which included all the patients in the analysis, was defined, in addition to specific sub-cohorts of patients based on diagnosis: an SLE cohort, a CTD cohort, an inflammatory arthritis cohort, a vasculitis cohort and a primary APS cohort; and cohorts of patients with certain characteristics of interest, including an Ro/La antibody cohort defined by patients with Ro/La antibodies and specific treatment cohorts defined by patients who took aspirin, low-molecular weight heparin (LMWH), CSs, conventional synthetic DMARDs (csDMARDs) or biologic DMARDs (bDMARDs) at any point during the pregnancy.

### Pregnancy outcomes

The outcome of the pregnancy was recorded as miscarriage, stillbirth or live birth. A cut-off of 24 weeks gestation was used to differentiate miscarriage from stillbirth [11]. Sex of the neonate, birth weight and mode of delivery were tabulated. Modes of delivery were classified as normal vaginal delivery, assisted delivery (forceps/ventouse), elective and emergency Caesarean section (EMCS), as recorded by the midwifery and obstetric teams.

### Maternal complications

Maternal, complications including infection (antenatal and post-partum), pre-eclampsia and PPH, were tabulated. A patient was considered to have PPH if estimated blood loss was >500 ml within 24 h after delivery [12].

### Fetal complications

Fetal complications, including sepsis, CHB, prematurity, low birth weight and neonatal intensive care unit (NICU) admission, were tabulated. Neonates born at ≤37 weeks of gestation were classified as being premature [13]. Neonates with a birth weight <2500 g were considered to have low birth weight [14].

### Statistical analysis

Descriptive statistics were used to summarize the data using R v.4.1.0 and RStudio v.1.4.1106. Comparisons of proportions were performed using χ^2^ tests, taking *P* < 0.05 as a threshold for significance.

### Ethics

No specific ethical approval was required or obtained for this analysis.

## Results

### Patient characteristics

Between January 2018 and January 2020, 98 women were seen in LIPS clinic antenatally and completed their pregnancy, excluding 17 women who were seen once in the LIPS clinic, deemed low risk and subsequently discharged back to their local rheumatology and obstetric services. Baseline characteristics are shown in Table 1. In terms of patient characteristics, the majority were of Caucasian ethnicity (*n* = 62, 63%), had a diagnosis of a CTD (*n* = 61, 62%), with SLE being the most common (*n* = 36, 37%), with stable disease during pregnancy (*n* = 86, 88%). Nearly one-third of patients had Ro autoantibodies (*n* = 31, 32%), and a quarter had aPL antibodies (*n* = 25, 26%). The majority of patients took aspirin during pregnancy (*n* = 61, 62%); about one-half took CSs and csDMARDs (*n* = 36, 47% and *n* = 47, 48%, respectively), and about one-third took LMWH (*n* = 32, 33%). Eight patients (8%) took a bDMARD, of whom two patients (2%) received tocilizumab and one received ofatumumab (1%) during pregnancy.

**Table 1:** Maternal characteristics of the lupus in pregnancy scanning cohort

Characteristic	*n* = 98
Age, median (IQR), years	32 (28–34)
Ethnicity, *n* (%)	
Caucasian	62 (63)
South Asian	24 (24)
East Asian	2 (2)
Afro-Caribbean	6 (6)
Mixed	4 (4)
Diagnosis, *n* (%)	
CTD	61 (62)
SLE	36 (37)
UCTD	7 (7)
SSc	6 (6)
SS	5 (5)
MCTD	4 (4)
Idiopathic inflammatory myositis	3 (3)
Inflammatory arthritis	15 (15)
Undifferentiated inflammatory arthritis	6 (6)
RA	5 (5)
JIA	3 (3)
PsA	1 (1)
Vasculitis	7 (7)
Granulomatosis with polyangiitis	3 (3)
Takayasu arteritis	2 (2)
Behçet’s disease	2 (2)
Primary APS	11 (11)
Unclear rheumatological condition	4 (4)
Disease activity, *n* (%)	
Stable disease during pregnancy	86 (88)
Uncontrolled disease during pregnancy	12 (12)
Antibody status, *n* (%)	
ANA	49 (50)
Ro	31 (32)
APS	25 (26)
La	14 (14)
Medications taken during pregnancy, *n* (%)	
Aspirin	61 (62)
Low-molecular weight heparin	32 (33)
CSs	36 (47)
csDMARDs	47 (48)
HCQ	31 (32)
AZA	21 (21)
Tacrolimus	4 (4)
MMF	2 (2)
SSZ	2 (2)
Mesalazine	1 (1)
CSA	1 (1)
bDMARDs	8 (8)
Etanercept	2 (2)
Adalimumab	2 (2)
Tocilizumab	2 (2)
Certolizumab	1 (1)
Ofatumumab	1 (1)

bDMARDs: biological DMARDs; csDMARDs: conventional synthetic DMARDs; IQR: interquartile range.

### Pregnancy outcomes of the LIPS cohort

Pregnancy outcomes for the LIPS cohort (*n* = 98) are shown in Table 2. Of the 98 completed pregnancies, 2 resulted in miscarriage (2%), 1 in stillbirth (1%) and 95 in live birth (97%). The median duration of gestation was 38 [interquartile range (IQR) 37–39] weeks, with a median birth weight of 3120 (IQR 2690–3410) g, and the most common modes of delivery were normal vaginal delivery (*n* = 34, 35%) and EMCS (*n* = 29, 30%). The most common maternal complications were PPH (*n* = 54, 56%) and infection (*n* = 21, 22%), with only one case of pre-eclampsia (1%). The most common fetal complications were prematurity (*n* = 22, 23%) and low birth weight (*n* = 16, 17%), with two neonates being affected by CHB (2%).

**Table 2:** Maternal and fetal outcomes for the lupus in pregnancy scanning cohort

Pregnancy outcome	LIPS cohort (*n* = 98)
Miscarriage	2 (2)
Deliveries	
Live birth	95 (97)
Stillbirth	1 (1)
Gestation, median (IQR), weeks	38 (37–39)
Sex of neonate	
Female	57 (59)
Male	39 (41)
Mode of delivery	
Normal vaginal delivery	34 (35)
Assisted delivery	19 (20)
Elective Caesarean section	14 (15)
Emergency Caesarean section	29 (30)
Neonatal birth weight, median (IQR), g	3120 (2690–3410)
Maternal complications	
Infection	21 (22)
Pre-eclampsia	1 (1)
Post-partum haemorrhage	54 (56)
Fetal complications	
Low birth weight (<2500 g)	16 (17)
Neonatal intensive care unit admission	6 (6)
Sepsis	1 (1)
Congenital heart block	2 (2)
Prematurity (<37 weeks)	22 (23)

IQR: interquartile range; LIPS: lupus in pregnancy scanning.

### Pregnancy outcomes by diagnosis, treatment or Ro/La sub-cohorts

Pregnancy outcomes for the sub-cohorts are shown in Tables 3–5.

**Table 3:** Maternal and fetal outcomes for the diagnosis sub-cohorts

Pregnancy outcome	LIPS cohort (*n* = 98)	SLE cohort (*n* = 36)	CTD cohort (*n* = 61)	Inflammatory arthritis cohort (*n* = 15)	Vasculitis cohort (*n* = 7)	Primary APS cohort (*n* = 11)
Miscarriage	2 (2)	1 (3)	1 (2)	1 (7)	0 0	0 0
Deliveries						
Live birth	95 (97)	35 (97)	59 (97)	14 (100)	7 (100)	11 (100)
Stillbirth	1 (1)	0 0	1 (2)	0 0	0 0	0 0
Gestation, median (IQR), weeks	38 (37–39)	38 (36–39)	38 (36–39)	38 (36–39)	39 (38–40)	37 (36–39)
Sex of neonate						
Female	57 (59)	24 (69)	36 (59)	10 (71)	3 (43)	5 (45)
Male	39 (41)	11 (31)	24 (41)	4 (29)	4 (57)	6 (55)
Mode of delivery						
Normal vaginal delivery	34 (35)	14 (40)	20 (33)	5 (36)	2 (29)	5 (45)
Assisted delivery	19 (20)	5 (14)	12 (20)	2 (14)	2 (29)	1 (9)
Elective Caesarean section	14 (15)	4 (11)	7 (11)	3 (21)	1 (14)	3 (27)
Emergency Caesarean section	29 (30)	12 (34)	21 (34)	4 (29)	2 (29)	2 (18)
Neonatal birth weight, median (IQR), g	3120 (2690–3410)	3206 (2690–3450)	3191 (2619–3460)	3098 (2555–3199)	3045 (2894–3408)	3054 (2770–3385)
Maternal complications						
Infection	21 (22)	10 (29)	16 (26)	2 (14)	2 (29)	1 (9)
Pre-eclampsia	1 (1)	1 (3)	1 (2)	0 0	0 0	0 0
Post-partum haemorrhage	54 (56)	18 (51)	31 (51)	8 (57)	5 (71)	8 (73)
Fetal complications						
Low birth weight (<2500 g)	16 (17)	5 (14)	11 (18)	3 (21)	0 0	2 (18)
Neonatal intensive care unit admission	6 (6)	4 (11)	4 (7)	2 (14)	0 0	0 0
Sepsis	1 (1)	0 0	1 (2)	0 0	0 0	0 0
Congenital heart block	2 (2)	0 0	2 (3)	0 0	0 0	0 0
Prematurity (<37 weeks)	22 (23)	10 (29)	15 (25)	4 (29)	0 0	3 (27)

IQR: interquartile range; LIPS: lupus in pregnancy scanning.

**Table 4:** Maternal and fetal outcomes for the treatment sub-cohorts

Pregnancy outcome	LIPS cohort (*n* = 98)	Aspirin cohort (*n* = 61)	LMWH cohort (*n* = 32)	CS cohort (*n* = 36)	csDMARD cohort (*n* = 47)	bDMARD cohort (*n* = 8)
Miscarriage	2 (2)	1 (2)	0 0	2 (6)	1 (2)	1 (13)
Deliveries						
Live birth	95 (97)	59 (97)	32 (100)	33 (92)	46 (98)	7 (88)
Stillbirth	1 (1)	1 (2)	0 0	1 (3)	0 0	0 0
Gestation, median (IQR), weeks	38 (37–39)	38 (36–39)	37 (35–37)	38 (35–39)	38 (36–39)	38 (37–39)
Sex of neonate						
Female	57 (59)	37 (63)	19 (59)	20 (61)	32 (70)	4 (57)
Male	39 (41)	22 (37)	13 (41)	13 (39)	14 (30)	3 (43)
Mode of delivery						
Normal vaginal delivery	34 (35)	24 (41)	12 (38)	14 (42)	16 (35)	2 (29)
Assisted delivery	19 (20)	13 (22)	4 (13)	6 (18)	8 (17)	1 (14)
Elective Caesarean section	14 (15)	7 (12)	8 (25)	5 (15)	8 (17)	1 (14)
Emergency Caesarean section	29 (30)	16 (27)	8 (25)	9 (27)	14 (30)	3 (43)
Neonatal birth weight, median (IQR), g	3120 (2690–3410)	3105 (3697–3388)	2886 (2587–3153)	2988 (2459–3385)	3120 (2685–3381)	3096 (2810–3170)
Maternal complications						
Infection	21 (22)	7 (12)	8 (25)	9 (27)	12 (26)	2 (29)
Pre-eclampsia	1 (1)	0 0	0 0	0 0	1 (2)	0 0
Post-partum haemorrhage	54 (56)	23 (39)	17 (53)	19 (58)	29 (63)	4 (57)
Fetal complications						
Low birth weight (<2500 g)	16 (17)	10 (17)	7 (22)	8 (24)	9 (20)	1 (14)
Neonatal intensive care unit admission	6 (6)	4 (68)	4 (13)	3 (9)	5 (11)	1 (14)
Sepsis	1 (1)	1 (2)	1 (1)	1 (3)	1 (2)	0 0
Congenital heart block	2 (2)	1 (2)	0 0	0 0	2 (4)	0 0
Prematurity (<37 weeks)	22 (23)	16 (27)	13 (41)	11 (33)	13 (28)	2 (29)

bDMARDs: biological DMARDs; csDMARDs: conventional synthetic DMARDs; IQR: interquartile range; LIPS: lupus in pregnancy scanning; LMWH: low-molecular weight heparin.

**Table 5:** Maternal and fetal outcomes for patients with Ro/La antibodies

Pregnancy outcome	LIPS cohort (*n* = 98)	Ro/La antibody cohort (*n* = 35)
Miscarriage	2 (2)	0 0
Deliveries		
Live birth	95 (97)	35 (100)
Stillbirth	1 (1)	0 0
Gestation, median (IQR), weeks	38 (37–39)	37 (35–39)
Sex of neonate		
Female	57 (59)	21 (60)
Male	39 (41)	14 (40)
Mode of delivery		
Normal vaginal delivery	34 (35)	13 (37)
Assisted delivery	19 (20)	5 (14)
Elective Caesarean section	14 (15)	3 (9)
Emergency Caesarean section	29 (30)	14 (40)
Neonatal birth weight, median (IQR), g	3120 (2690–3410)	3147 (2478–3460)
Maternal complications		
Infection	21 (22)	7 (20)
Pre-eclampsia	1 (1)	0 0
Post-partum haemorrhage	54 (56)	23 (66)
Fetal complications		
Low birth weight (<2500 g)	16 (17)	8 (23)
Neonatal intensive care admission	6 (6)	3 (9)
Sepsis	1 (1)	0 0
Congenital heart block	2 (2)	2 (6)
Prematurity (<37 weeks)	22 (23)	12 (34)

IQR: interquartile range; LIPS: lupus in pregnancy scanning.

### Maternal characteristics of neonates affected by CHB

Two neonates were affected by CHB. In both cases, the maternal diagnosis was SS, and both mothers had ANA, Ro and La antibodies; stable disease during pregnancy was maintained with HCQ taken during pregnancy. In one case, the mother also took CSs during pregnancy; in the other case, the mother also took aspirin during pregnancy.

### Maternal characteristics of patients affected by pre-eclampsia

One patient was affected by pre-eclampsia. In the affected case, the maternal diagnosis was SLE. The mother had ANAs, and she received HCQ, LMWH and prednisolone 20 mg daily during pregnancy. She did not receive aspirin during pregnancy. She had previously received two rituximab infusions 1 year before her pregnancy. Unfortunately, she discovered she was pregnant only at 26 weeks gestation, by which point she had already received four monthly infusions of CYC at approximately 12-, 16-, 20- and 24 weeks of gestation. No further CYC infusions were given after her pregnancy was confirmed, and she was initiated on LMWH and HCQ. She subsequently had an EMCS at 31 weeks, with the baby being discharged successfully from the NICU after 8 days with no obvious disability.

### Maternal characteristics and pregnancy outcomes in patients receiving tocilizumab and ofatumumab

Two patients received tocilizumab during pregnancy.

In the first case, the maternal diagnosis was Takayasu arteritis, and the mother had no autoantibodies. Initially, she received methylprednisolone with two cycles of CYC, which did not control her disease effectively. She was subsequently commenced on tocilizumab, which stabilized her condition 2 years before her pregnancy. During her pregnancy, she continued to receive tocilizumab, prednisolone at an initial dose of 20 mg daily, which was reduced and maintained at 7.5 mg daily during the third trimester, AZA, LMWH and aspirin. She received tocilizumab throughout her pregnancy until delivery. The outcome was a live birth at 39 weeks via EMCS, with a birth weight of 2768 g, complicated by PPH.

In the second case, the maternal diagnosis was JIA. The mother had ANAs, and she received HCQ and prednisolone 4 mg daily and tocilizumab during pregnancy. The outcome was early miscarriage at 7 weeks of gestation.

One patient received ofatumumab during pregnancy. In this case, the maternal diagnosis was SLE. The mother had ANAs and Ro antibodies, and she received AZA, prednisolone, aspirin, LMWH and three ofatumumab infusions in the second trimester during pregnancy. A decision was made, owing to high disease activity during pregnancy that threatened maternal and neonatal wellbeing, to give ofatumumab during the second trimester of pregnancy, which resulted in successful B-cell depletion, disease control and tapering of her CS dose from 30 mg in the first trimester to 5 mg in the third trimester. The outcome was a live birth via EMCS at 36 weeks, with a birth weight of 2852 g, complicated by PPH and maternal infection, in addition to a NICU admission for the neonate. The maternal infection was a minor urinary tract infection, which was managed with oral antibiotics. After a 9-day NICU admission, the baby was discharged successfully with no obvious disability.

### Maternal characteristics in pregnancies ending in miscarriage and stillbirth

Two pregnancies ended in miscarriage. In the first case, the maternal diagnosis was SLE. The mother had ANAs, and she took HCQ, prednisolone 5 mg daily and aspirin during pregnancy. The miscarriage took place at 13 weeks of gestation. The second case ending in miscarriage was described above, with a maternal diagnosis of JIA and receiving tocilizumab during pregnancy. The miscarriage took place at 7 weeks of gestation.

One pregnancy ended in stillbirth at 25 weeks of gestation. In the affected case, the maternal diagnosis was idiopathic inflammatory myositis. The mother had Jo-1 antibodies, and she received prednisolone 10 mg daily during pregnancy. The mother had stable disease activity during pregnancy.

### Maternal characteristics in pregnancies complicated by PPH

Fifty-four pregnancies were affected by PPH (56%). The maternal characteristics in affected patients are shown in Table 6. Of the 54 pregnancies affected by PPH, aspirin was taken in 33 (61%) and LMWH in 17 (31%).

**Table 6:** Maternal characteristics of pregnancies complicated by post-partum haemorrhage

Characteristic	PPH cohort (*n* = 54)
Diagnosis, *n* (%)	
CTD	31 (57)
SLE	18 (33)
UCTD	3 (6)
SSc	2 (4)
SS	4 (7)
MCTD	3 (6)
Idiopathic inflammatory myositis	1 (2)
Inflammatory arthritis	8 (15)
Undifferentiated inflammatory arthritis	4 (7)
RA	2 (4)
JIA	1 (2)
PsA	1 (2)
Vasculitis	5 (9)
Granulomatosis with polyangiitis	2 (4)
Takayasu arteritis	1 (2)
Behçet’s disease	2 (4)
Primary APS	8 (15)
Unclear rheumatological condition	2 (4)
Disease activity, *n* (%)	
Stable disease during pregnancy	50 (93)
Uncontrolled disease during pregnancy	4 (7)
Antibody status, *n* (%)	
ANA	29 (54)
Ro	22 (41)
APS	15 (28)
La	9 (17)
Medications taken during pregnancy, *n* (%)	
Aspirin	33 (61)
LMWH	17 (31)
CSs	19 (35)
csDMARDs	29 (54)
HCQ	19 (35)
AZA	10 (19)
Tacrolimus	2 (4)
MMF	2 (4)
SSZ	1 (2)
Mesalazine	1 (2)
bDMARDs	4 (7)
Adalimumab	1 (2)
Tocilizumab	1 (2)
Certolizumab	1 (2)
Ofatumumab	1 (2)

bDMARDs: biological DMARDs; csDMARDs: conventional synthetic DMARDs; LMWH: low-molecular weight heparin; PPH: post-partum haemorrhage.

## Discussion

The importance of the multidisciplinary team in the management of high-risk pregnancies in patients with SLE and other rheumatological conditions is well established [3, 15]. However, in clinical practice, there are a variety of service models that implement multidisciplinary teams in different ways. Our study highlights positive patient outcomes that have resulted from a unique service model to care for high-risk pregnancies in patients with SLE and other rheumatological conditions.

The cohort of patients seen within the LIPS clinic were found predominantly to be Caucasian women >30 years of age, diagnosed with a CTD (mostly SLE), with stable disease throughout pregnancy. However, interestingly, there was also a significant proportion of ethnic minorities (34%) in this cohort, which reflects the population diversity seen in the North West of England, where the clinic is located.

The pregnancy outcomes found within this cohort were notable for having a high live birth rate (97%) and very low rates of miscarriage and stillbirth (2% and 1% respectively), especially compared with previously reported miscarriage rates of 7% and reported stillbirth rates of 5–16% in patients with SLE [16]. One explanation for this finding is that patients followed up in the LIPS clinic through to delivery are optimized in terms of disease activity and treatment to minimize risks of miscarriage (e.g. with aspirin and LMWH) [17]. Alternatively, our data might underestimate miscarriage rates because early miscarriages might occur before a patient can be referred and seen in the LIPS clinic.

In terms of maternal complications, an interesting finding from our analysis was the high rate of PPH (>500 ml; 56%) compared with published population incidence rates of 34% [18]. This might be attributable to the high proportion of patients within our cohort who took concurrent aspirin and LMWH (62% and 33%, respectively). Furthermore, 30% of patients in the LIPS cohort underwent EMCS, which has been associated with higher rates of haemorrhage, compared with the rate of 17% of EMCS reported by National Health Service (NHS) Maternity statistics [19, 20]. A higher rate of EMCS coupled with the increased rate of aspirin and LMWH use might explain the increased rate of PPH seen in the LIPS cohort.

Furthermore, we found a high rate of minor maternal infections (22%), where minor infections were defined as infections that responded to oral or i.v. antibiotics and did not cause significant organ dysfunction requiring admission to intensive care. The high rates of infection could be explained, in part, by the high proportion of patients within our cohort who took CSs and csDMARDs (47% and 48%, respectively), which will cause varying degrees of immunosuppression and increased susceptibility to infection. Furthermore, rates of minor infection might be underestimated, because patients could have received treatment from primary care and not subsequently informed the LIPS clinic.

Although we found several positive outcomes in our cohort compared with other studies in similar cohorts, we still found increased proportions of adverse pregnancy outcomes, such as prematurity, EMCS rates and low birth weights, compared with outcomes reported in healthy women [3]. For example, prematurity in our cohort was found in 23% of live births, whereas NHS Maternity statistics report prematurity rates of 6.3% [20]. Likewise, we reported rates of EMCS and low birth weight of 30% and 17%, respectively, that are higher than the NHS Maternity statistics, which report rates of 17% and 7%, respectively [20].

Previous studies have found higher rates of EMCS, prematurity and low birth weight in patients with inflammatory arthropathies and CTDs [21–26]. Therefore, our findings are in keeping with previously published findings, suggesting that patients with IRDs have increased risk of APOs.

Autoimmune CHB is an immune-mediated disease that is attributed to the transplacental passage of Ro/La antibodies, which has been reported to occur in ∼2% of pregnancies in women with Ro/La antibodies [27]. In terms of pharmacological prophylaxis to reduce the risk of CHB, HCQ initiated early in pregnancy has been shown to reduce the risk of CHB if started towards the end of the first trimester [27]. Interestingly, the LIPS cohort in our study included 31 patients with Ro antibodies (32%), of which 2 pregnancies were complicated by neonatal CHB (2 of 31, 6.5%) despite the use of HCQ during pregnancy. This reaffirms that the risk of CHB remains an important consideration when managing patients with IRDs antenatally.

Prescribing of bDMARDs antenatally remains a complex consideration, because many drugs used to minimize disease activity in IRDs are not licensed in pregnancy. Although registry studies have suggested that certain bDMARDs, predominantly based on data in patients taking TNF-α inhibitors (TNFi) for the treatment of RA suggest that TNFi are relatively safe in pregnancy, there is a lack of observational data for newer agents, such as tocilizumab, to assess safety in pregnancy [28, 29]. Interestingly, eight patients in the LIPS cohort received bDMARDs during pregnancy, with five receiving TNFi, two receiving tocilizumab and one receiving ofatumumab. Especially interesting are the patients who received tocilizumab and ofatumumab, because the current recommendations are to discontinue these agents in the first trimester and to use them with caution later in pregnancy [30]. One of the patients receiving tocilizumab had a miscarriage, whereas the other had a live birth with minimal complications. The patient who received ofatumumab had a successful live birth that was complicated by APOs, which are likely to be more related to significant disease activity rather than a consequence of the medication itself. Further observational studies will help to clarify the safety of these agents in pregnancy.

Another point to consider is the referral criteria for our service. Unlike some other service models, the LIPS clinic accepted referrals from any health-care professional for any patient with a high risk rheumatological conditions, whereas other clinics often predominantly see patients with SLE. This model recognizes that patients across the autoimmune disease spectrum will have increased risks of APOs and might benefit from specialist opinion in the form of a joint obstetric/rheumatology clinic. Furthermore, this also allows patients who have not yet had a confirmed diagnosis or who have complicated overlap syndromes to receive specialist care that might improve their pregnancy outcomes.

When comparing our findings with published data from clinics using similar service models in Sheffield and London, we found that our live birth rates are significantly higher than the results from the Sheffield clinic (28 of 41 *vs* 102 of 105, Sheffield *vs* Manchester) and comparable to results from the London clinic (99 of 102 *vs* 102 of 105, London *vs* Manchester) [31, 32].

In conclusion, we report favourable pregnancy outcomes resulting from a unique service model involving a joint obstetric and rheumatology clinic in a tertiary centre. The benefits of this model might be applied to other centres, although further studies and longitudinal data would help to improve clinical care further for high-risk pregnancies in patients with IRDs.
